# Books and Magazines

**Published:** 1901-12

**Authors:** 


Nothnagel's Encyclopedia of Practical Medicine.—Edited by Alfred Stengel, M. D., Professor of Clinical Medicine in the University of Pennsylvania, Visiting Physician to the Pennsylvania Hospital.

W. B. Saunders & Co., the publishers, announce the early appearance of an American edition of this great work, "translated by men possessing thorough knowledge of both English and German." Each volume will be edited by a prominent specialist on the subject to which it is devoted. It will thus be brought thoroughly up-to-date.

The publishers state that the usual method of publishers, when issuing a work of this kind, has been to compel physicians to take the entire system. This seems to us in many cases to be undesirable. Therefore, in purchasing this encyclopedia, physicians will be given the opportunity of subscribing for the entire system at one time, but any single volume or any number of volumes may be obtained by those who do not desire the complete series. This latter method, while not so profitable to the publisher, offers to the purchaser many advantages which will be appreciated by those who do not care to subscribe for the entire work at one time.

This American edition of Nothnagel's Encyclopedia will, without question, form the greatest system of medicine ever produced, and the publishers feel confident that it will meet with general favor in the medical profession.

BLAKISTON'S PHYSICIANS' VISITING LIST for 1902 is received. This is the fifty-first year of its publication. It is too well known to the medical profession to require more at our hands than an announcement that the 1902 edition is ready. For twenty-five patients per day or week, $1.00; fifty patients, $1.25. P. Blakiston's Sons & Co., 1012 Walnut Street, Philadelphia.

